# Treatment-resistant hiccups during general anesthesia possibly caused by remimazolam: a case report

**DOI:** 10.1186/s40981-024-00714-3

**Published:** 2024-05-24

**Authors:** Miku Sakurai, Yusuke Matsui, Tomonori Takazawa, Yoji Kabasawa, Wataru Nagumo, Ryo Takada, Shigeru Saito

**Affiliations:** 1 Department of Anesthesiology and Intensive Care, Gunma University Hospital, 3-39-15 Showa-machi, Maebashi, 371-8511 Japan; 2 Department of Anesthesiology, Faculty of Medicine, University of Toyama, 2630 Sugitani, Toyama, 930-0194 Japan

**Keywords:** Hiccups, Remimazolam, Benzodiazepine

## Abstract

**Background:**

Previous reports have described hiccups during general anesthesia that were possibly induced by drugs, including benzodiazepines. However, there are few reports of hiccups caused by remimazolam.

Case presentation

A 75-year-old woman underwent corneal transplantation under general anesthesia with remimazolam. She presented with hiccups once the effects of muscle relaxants used during induction wore off, which persisted even after various treatments, such as the administration of antipsychotic drugs. However, when remimazolam administration was terminated after surgery to awaken the patient, the hiccups stopped and did not recur after extubation. Evaluation of predicted blood levels of remimazolam suggests that higher levels of remimazolam might cause hiccups.

**Conclusion:**

Remimazolam might induce hiccups during general anesthesia. Anesthesiologists should consider administering muscle relaxants or changing the anesthetic in cases of refractory hiccups under general anesthesia.

## Background

Drug-induced hiccups have been reported in the past, with benzodiazepines being identified as one of the possible causes [1]. Hiccups during general anesthesia and their possible treatment have also been previously reported [2, 3]. A randomized controlled trial comparing remimazolam and propofol regarding their safety and efficacy during sedation for gastric endoscopy in elderly patients suggested a relatively high incidence of hiccups (> 10%) in the remimazolam group patients [4]. To the best of our knowledge, however, hiccups during general anesthesia with remimazolam have not been reported.

## Case presentation

**Fig. 1:**
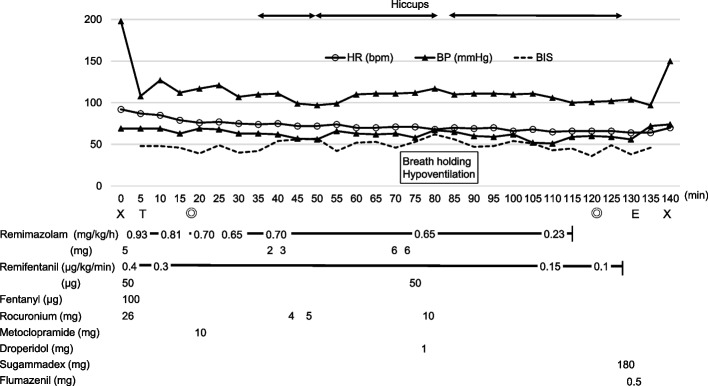
Anesthesia record. Hiccups occurred approximately 35 min after administration of rocuronium 26 mg, which were interrupted by bolus infusion of rocuronium but resumed as its effects wore off. After remimazolam administration was terminated, the hiccups ceased without administering muscle relaxants. The Xs in the figure indicate the beginning and end of anesthesia, the double circles indicate the beginning and end of surgery, and T and E indicate the timing of intubation and extubation, respectively

A 75-year-old woman (weight, 44 kg; height, 137 cm) was scheduled for corneal transplantation under general anesthesia. Preoperative evaluation indicated anemia and hypertension. Anesthesia was induced with remimazolam, remifentanil, and rocuronium and was maintained with remimazolam and remifentanil (Fig. 1).

Her vital signs remained stable, and no unusual events occurred in the immediate post-induction period and after commencement of surgery. However, she began to hiccup approximately 15 min after the start of surgery (approximately 35 min after the administration of muscle relaxants). Initially, mistaking the hiccups as small spontaneous breaths, the anesthesiologists increased the remimazolam dosage, but the hiccups continued. Gradually, the hiccups became more robust, and the anesthesiologists realized that it was not spontaneous breathing but hiccups. At this time, the anesthesiologist asked the surgeon if the hiccups would interfere with the surgery, but it was determined that they would not be a problem. Although bispectral index (BIS) values ranged from 49 to 60, which is appropriate for general anesthesia, an additional 6 mg of remimazolam was administered to eliminate the possibility of insufficient anesthesia depth. Nevertheless, the hiccups persisted. In addition to the 10-mg metoclopramide that had already been administered as an antiemetic, other measures were taken, including breath holding, carbon dioxide sequestration, and 1-mg droperidol administration. However, all of these treatments were ineffective. Further, the hiccups gradually became more prominent as the effects of the muscle relaxants wore off. Additional rocuronium doses were only able to temporarily suppress the hiccups, as long as the muscle relaxant effects lasted. This situation continued until the surgery was completed.

Fifteen minutes after the termination of remimazolam administration for extubation, the hiccups abruptly stopped. The patient was then awakened and extubated after 0.5-mg flumazenil administration. After extubation, the patient expressed that she had experienced no adverse events, such as intraoperative arousal. Her postoperative status was unremarkable, and she was discharged 6 days postoperatively, as scheduled.

## Discussion

In the present case, despite various treatments for intraoperative hiccups, all of which were ineffective, the hiccups finally disappeared when the patient was awakened from general anesthesia.

The possible causes of hiccups under general anesthesia include the adverse effects of drugs, physical stimulation by the endotracheal tube, insufficient anesthesia depth, and surgical procedures [5]. In this case, we assumed that the benzodiazepine remimazolam was the cause of the hiccups, because they disappeared after the termination of remimazolam administration. Since we administered rocuronium immediately after remimazolam administration at the time of anesthesia induction, development of hiccups in the early course of anesthesia might have been masked. Remimazolam was reported to cause more hiccups than propofol in sedation for endoscopy without muscle relaxants, supporting our hypothesis [4]. We considered it unlikely that stimulation by the endotracheal tube was the cause of the hiccups since they disappeared before extubation. Further, insufficient anesthesia was an unlikely cause of the hiccups because her BIS values suggested appropriate anesthesia depth, and additional anesthetic administration was ineffective for terminating the hiccups. Since vagus nerve stimulation, such as by application of ocular pressure, is one of the suggested treatments for suppressing hiccups, and since the hiccups continued even after the surgery was completed, we considered it unlikely that surgical stimulation was the cause of the hiccups.

**Fig. 2:**
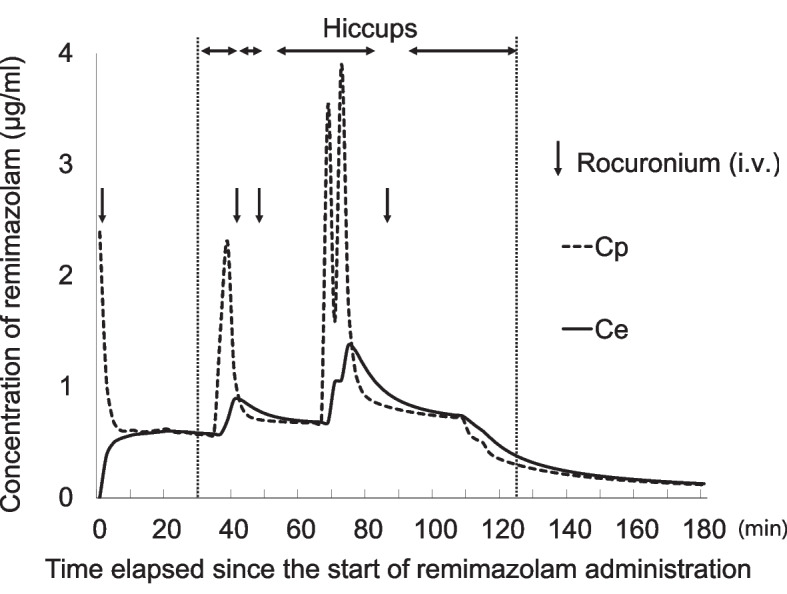
The course of hiccups and the predicted plasma and effect site concentration of remimazolam. The hiccups continued at the time of high remimazolam concentrations, except immediately after the administration of rocuronium. The hiccups stopped after the estimated remimazolam concentration decreased. Cp, predicted plasma concentration; Ce, effect site concentration

To support our hypothesis that the cause of the hiccups could be remimazolam, we retrospectively estimated the patient’s remimazolam concentrations using a recently published pharmacokinetic model [3]. The simulated remimazolam concentration results and the course of the hiccups are shown in Figure 2. Remimazolam concentration was maintained from the time of anesthesia induction until the end of the administration, which could be inferred as a sufficient anesthesia depth. At no time was the predicted effect site concentration of remimazolam during anesthetic administration lower than at the point at which hiccups finally stopped. Although it is difficult to rule out the possibility that other drugs, including fentanyl and remifentanil, might have been the cause of the hiccups, based on the timing of drug administration and hiccup cessation, as described above, and previous reports of medications prone to causing hiccups, we believe that remimazolam was the most likely cause.

In this case, stimulation of the nasopharynx with cold water and eye compression, which have been reported as effective treatments for hiccup termination, was difficult to perform because of the proximity to the surgical field. Another effective way to stop hiccups is to administer muscle relaxants. Indeed, the administration of rocuronium interrupted the hiccups, albeit temporarily, in this case. If muscle relaxants are contra-indicated due to the need for intraoperative neuromonitoring or other reasons, chlorpromazine can be used to stop intraoperative hiccups [6]. Alternatively, changing to another anesthetic might be effective in cases of remimazolam-induced hiccups.

## Data Availability

Data relevant to this case report are not available for public access because of patient privacy concerns but are available from the corresponding author on reasonable request.

## Abbreviation

BIS: Bispectral index
